# The Evolution of Community Benefit: Perspective on Progress Toward Purpose

**DOI:** 10.3389/fpubh.2020.00027

**Published:** 2020-03-02

**Authors:** Connie J. Evashwick, Penrose Jackson

**Affiliations:** ^1^Independent Consultant, Del Mar, CA, United States; ^2^Vermont Public Health Institute, Burlington, VT, United States

**Keywords:** community benefit, non-profit hospitals, tax-exemption, hospitals and community engagement, hospital tax-exempt policy, IRS, hospital finance

## Abstract

To maintain exemption from federal taxes, non-profit hospitals in the USA are required to contribute to their communities an amount comparable to the taxes they otherwise would have paid. Since 2008, non-profit hospitals have had to file Form 990 Schedule H with the Internal Revenue Service (IRS) to document their “Community Benefit” (CB) activities. The purpose of this article is to present an overview of the evolution of hospitals' engagement with their communities and to examine how the policy enforced by the IRS has evolved. The IRS has not made explicit the assumptions underlying the CB policy. As a result, the evidence about the impact of CB policy and CB activities on the health of a community is sparse. Non-profit hospitals are spending millions of dollars in CB activities and reporting requirements annually, but if and how these expenses contribute to a community's health and well-being are unclear. Conceptual frameworks, such as logic models or Collective Impact models, could be used to explicate the assumed relationships. As the field has evolved and grown more complex, identifying and measuring the contributions of a single hospital or single program to the health status of a community have become more challenging. Collaboration—promoted by the IRS and CDC—has increased these challenges. Until assumptions about relationships are made explicit and tied to measurable goals, non-profit hospitals must continue to comply with IRS requirements but should use their own targets, metrics, and evaluations to ensure that the resources devoted to CB programs are being used cost-effectively.

Community Benefit, as defined by the Internal Revenue Service (IRS), represents what non-profit hospitals contribute to their communities in lieu of paying federal taxes. Because the IRS has never articulated the assumptions upon which the community benefit requirement is based, nor desired outcomes, the regulation cannot be evaluated nor validated. Without a rationale and explicit goals or an underlying causal model, it is difficult ascertain how or if hospital community benefit activities have impacted individuals, communities, or institutions commensurate with the costs incurred. The leap to the ultimate question is also challenging: is the policy of requiring non-profit hospitals to provide community benefit as defined by the IRS improving the health status of the communities served? Or, are hospitals simply fulfilling a financial obligation by spending money on a variety of activities that the IRS deems “contribute” to the community?

This brief review of the evolution of community benefit demonstrates that the assumptions underlying the IRS regulation were not delineated in a way that supports a rigorous approach to testing and evaluation. The intent of this paper is not to describe theories that could lead to evaluation methodology that would provide statistical outcome measures or to lead us to an evidence-based conclusion about the impact to date of community benefit activities. Rather, we intend to show that the community benefit regulation was implemented without the benefit of theories related to health behavior, measurable goals and objectives derived from health management, relationships expressed in Collective Impact models, or metrics for measuring impact on community health status. Without theoretical and statistical rigor, community benefit cannot be expected to succeed in changing the health status of a community or provide evidence about what does or does not work. By understanding the evolution of community benefit, we may find a way forward to make the policy effective in improving the health status of a community.

## Background and Brief History of Hospitals and Community Benefit

The history of the hospital's role in the community dates back hundreds of years to ancient civilizations in Mesopotamia, Egypt, Greece, and Rome. Not until the latter part of the twentieth century were US hospitals pressured to document contributions to the communities they serve. The requirement for hospitals to engage in activities to benefit their community in exchange for exemption from federal taxes was first articulated in 1969 to the American Hospital Association (AHA) by the IRS as Revenue Ruling 69-645, 1969-2. CB 117. It was neither legislation nor regulation—simply a letter of opinion. For decades, the AHA and its members argued that hospitals contribute to their communities in many ways, saying it was unnecessary to quantify their contributions. The AHA produced a document referred to as the “Gold Standard of Community Benefit,” authored by Sigmond (1). While the “Gold Standard” was written, circulated, and discussed, it was never formally adopted by the AHA Board of Governors. Nonetheless, it provided guidance to hospitals on how they should interact with the communities they serve.

Although the AHA has focused on the issues deemed most salient by its members, which tend to be internal operations, it has always recognized hospitals' efforts to engage with their communities. For example, the AHA collaborated with the Robert Wood Johnson Foundation on Hospital Initiatives in Long-Term Care (2), an initiative focused on health systems demonstrating how they could expand their work with community providers to offer a broader scope of services to seniors and to those with complex, chronic problems. Also, the (then) Hospital Research and Educational Trust (HRET), AHA's research/demonstration arm, created the Office on Aging and Long-Term Care in 1981 (3). The advent of the prospective payment system by Medicare in 1982 gave visibility to this new unit, as it provided marked incentives for hospitals to work with seniors and the community-based services that helped prevent re-hospitalization. The AHA launched the Foster G. McGaw Award (4) and the Dick Davidson Nova Award (5), both designed to recognize health systems and individuals who are leaders in community engagement. They also created Community Connections, an annual compilation of succinct descriptions of hospital interactions with their community (6), which was maintained from 2005 to 2016.

In the 1980's the Catholic Health Association (CHA) countered challenges that hospitals and other health-related entities should not be exempt from taxes merely because of their religious affiliation. CHA initiated the “Social Accountability Budget” (7), which identified and categorized the types of hospital activities benefitting their communities. CHA also contracted with an accounting firm to develop an information system to report activities in ways that could be translated into quantifiable amounts and dollar values. The Community Benefit Inventory for Social Accountability (CBISA) evolved into the first management information system for reporting community benefit (8).

Also during the late 1980's, California's Public Health Institute conducted a national demonstration project on *Advancing the State of the Art of Community Benefit* (9). The project report provided guidance for structure and organization and identified key challenges of program planning and implementation.

Later during the 1990's, the American College of Healthcare Executives (ACHE), whose members are individual healthcare managers holding a range of administrative positions in hospitals and healthcare organizations, offered its perspective on community engagement. An ACHE policy statement on “the role of the healthcare executive within the community” (10) was first introduced in 1989 and has subsequently been revised several times (most recently in 2016). This policy articulates the rationale for healthcare executives to work with their communities, based on ethics rather than financial gain. In 1999, two ACHE senior executives conducted a study of hospital activities in their communities. The report of their exploration and interviews with key leaders is reported in the book, *Achieving Success Through Community Leadership* (11). The range of activities reported and the commitment of the leaders made clear the importance to health systems and hospitals of working with the community. “Lessons learned” were summarized; however, no quantifiable metrics were set forth as global or common goals for healthcare executives to pursue.

During the late 1980's and through the 1990's, several states joined in the crusade to require that hospitals document their involvement with their communities in order to maintain exemption from state taxes. California, Washington, Texas, and Illinois were among the leaders to advance strict provisions. The State of California, under SB697, requires goals and quantifiable impacts, including measurable objectives to be achieved within specified timeframes (12). However, since each state implemented its own requirements, these laws provide no multi-state consistency (13) about the quantifiable goals of community benefit.

In the 2000's, Iowa's Senator Grassley challenged hospitals about whether their contributions to their communities warranted their tax-exempt status. He convened hearings, and the resultant visibility raised the issue to such importance that the US Government Accountability Office (GAO) and Congressional Budget Office (CBO) examined the issues related to community benefit and, respectively, produced notable reports (14, 15). In 2007, primarily as a result of the momentum created by Senator Grassley, the IRS added Schedule H to Form 990 and required that this be completed by all non-profit hospitals that desired to maintain their federal tax exemption. The reporting form was phased in, starting in 2008, with complete reporting required in 2009. Note that for-profit hospitals and government hospitals, as well as all other types of healthcare facilities, do not need to file Schedule H nor explain if or how they benefit the community.

In its rule-making for reporting community benefit on Form 990, the IRS adopted CHA's Social Accountability approach and used the definitions and categories from CBISA. As noted above, CBISA (and therefore Form 990) catalogs hospitals' efforts to contribute to their communities, with the inherent assumption that these activities would result in improving community health. Note that the majority of community benefit funds−85% by some accounts–are spent on charity care and uncompensated clinical care (16), leaving relatively few dollars to spread across a fairly wide range of internal and community-oriented activities.

Passed in 2010, the Affordable Care Act (ACA) required that non-profit hospitals conduct triennial community health needs assessments (CHNAs). The first full round was completed in 2012–13. The ACA also required that hospitals promulgate Implementation Strategies to describe how they would address needs identified in the most recent CHNA. By 2019, all non-profit hospitals wishing to maintain their exemption from federal taxes have reported at least two rounds of CHNAs and Implementation Strategies. Some non-profit hospitals have begun aligning Implementation Strategy goals specific to their institution with the Community Health Improvement Plan (CHIP) created by the local public health department or other local health and social service agencies.

Despite all of these efforts by the IRS as well as the hospital industry, no standardized short-term goals or long-term quantifiable impacts are required by non-profit hospitals in performing or reporting community benefit for federal purposes. Part VI of Schedule H does ask the reporting hospitals to comment on how well activities have worked in meeting community need, and Implementation Strategies are expected to include measures. However, the direct relationship between given activities and the health status of the community or a sub-set of the community need not be reported with a specific format or metric. Most hospitals have excellent quality and clinical metrics, but connecting these with community measures has been problematic. Hospitals invest community benefit dollars in programs and processes that often have well-documented value but are not usually measured in concert with cross-community investments and collaborations.

## Models, Frameworks, and Evidence

Parallel to the practitioners' efforts to implement community benefits and report their expenses to the IRS, researchers and academicians working since the early 1970's in public health, healthcare management, and community sociology have advanced ways to think about the health system of a community and the health status of its members. The models presented here are just two of many having the potential to further the analysis of community benefit in an evidence-based way to improve the measurable health status of a community.

**Figure 1:**
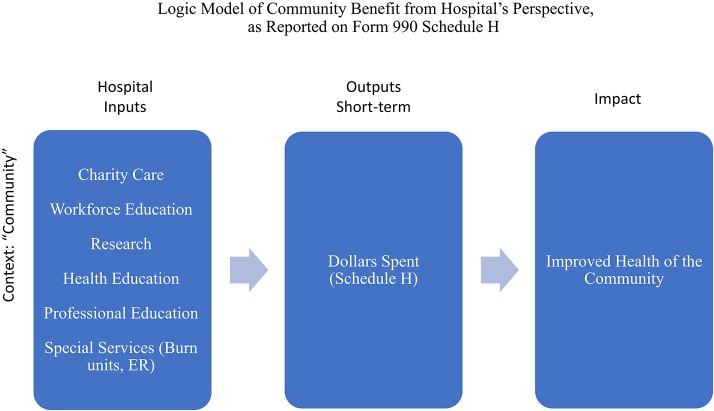
Logic model for hospital community benefit, reflecting the position of the IRS in 2007.

A “logic model” is a tool for guiding program evaluation (17). It has been embraced by the CDC to evaluate public health grant proposals to demonstrate that proposed interventions will succeed in accomplishing the changes or improvements in health behavior that the research proposes to investigate or that the implementation aims to achieve. The basic components of a logic model are Inputs (e.g., hospital, staff, community), Outputs (e.g., the types of activities performed by the hospital, the number of people participating, the number of procedures done—“process” measures), and the Outcomes, broken into Short-Term or Proximal (immediate outcomes), Middle-Term (more significant changes in behavior, policy, and community patterns), and Long-Term or Distal (sustained change at all levels). For the first fifty years, from 1969 to 2009, the description of community benefit was primarily only of outputs—numbers of activities, number of participants, number of dollars spent. Figure 1 shows how the IRS requirements as expressed in 2007 would be displayed graphically in an elementary logic model.

Over time, the IRS and ACA began to add a degree of rigor to the flow of activities and expectations by requiring a community health needs assessment, selection of priorities based on needs, an implementation strategy, and an evaluation of interventions related to previously identified needs.

**Figure 2:**
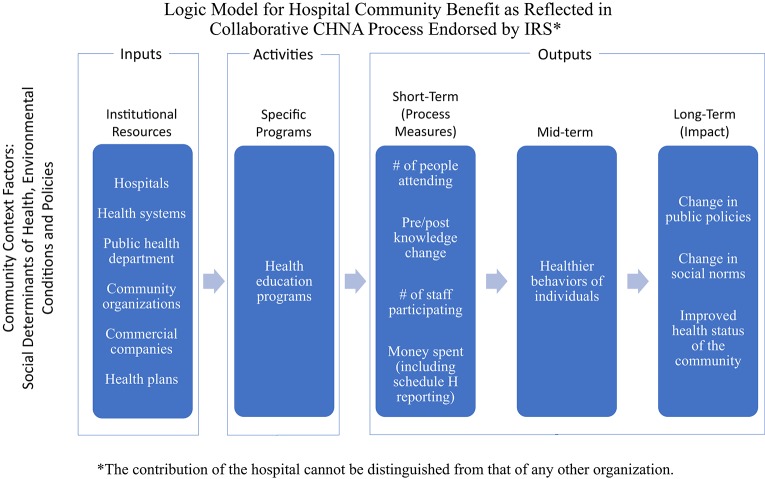
Logic model for community benefit showing multiple agency involvement.

Collaboration among community organizations has been encouraged, particularly for conducting the CHNA. Multi-sector collaboration reflects recognition of the relevancy of social determinants of health (SDOH) to the health of a community, as organizations with different perspectives, such as housing and transportation, become involved with organizations primarily focused on health, including hospitals and public health departments. However, promoting such collaboration in general deters measurement of the contributions of any one organization. Figure 2 shows a revised logic model with various organizations involved in affecting the health status of the community.

Those who work in research, program evaluation, or evidence-based program administration will quickly see that the logic model—which is much more explicit than the IRS ever explained–is nonetheless fraught with vagaries that undercut the documentation of any given program's impact on a community's health. When several hospitals work together on a community's CHNA, and then develop individual Implementation Strategies, sorting out the contribution of any one hospital becomes all the more challenging.

Collective Impact (CI) is a more recent framework that recognizes the complexities of a community's health by acknowledging that no institution can single-handedly change the health status of a given community (18, 19). The CI model has risen to prominence over the past decade, concomitant with the world-wide recognition of the importance of the social determinants of health. Using CI to address SDOH formalizes the collaborative effort of community organizations taking a multi-faceted approach that considers health assets, housing, transportation, food supply, employment, education, among other life facets. The CI model has five principles of participation—including shared goals, consensus on outcome measures, and constant communication. One principle is support by a “backbone institution,” often the community's hospital. The benefit and drawback of a CI approach is that no single institution—including the hospital in the role of the backbone institution–can take full credit for a change in the health conditions or health status of a community, as the model acknowledges that change in social fabric is not the product of a single entity's actions, but the combined actions of all partners.

Results-Based Accountability (RBA) is a powerful tool many CI initiatives use in evaluating progress. Developed by Friedman in the 1980's, RBA is today held by the Fiscal Policy Studies Institute, which defines it as “a disciplined way of thinking and taking action that communities can use to improve the lives of children, youth, families, adults and the community as a whole” (20).

The essence of RBA is contained in three questions:

- How much did we do?
- How well did we do it?
- Is anyone better off?

All contributors to a CI initiative can use the RBA framework to evaluate the value of their individual efforts to the overall goal. This method enables quantifiable measures of activities to be related to quantifiable measures of results. The final question—is anyone better off—can be used as a proxy for improvement in community health status.

Both a refined logic model and a CI model can be used to document the impact of the community benefit activities of a given hospital—although the extent to which credit for change can be claimed by a single hospital can be challenged. Other models could also be used to move community benefit to an evidence-based approach, such as the RE-AIM model used by the public health field to focus on program evaluation and the Precede-Proceed model used in health education.

## Discussion

In 2009, not long after reporting of Schedule H began, a pre-conference to Academy Health was held to consider questions related to the impact of the new regulation. The conference, “Community Benefit: Moving Forward with Evidence-Based Policy and Practice,” (21) called for rigorous evaluation and a research agenda. The mandate was largely ignored. The IRS engaged the CDC to convene a workshop in 2011 on issues related to community benefit implementation and measurement, but the results were not widely shared with either the practice or the research community.

The Health Research and Educational Trust (HRET) unit of the American Hospital Association compiled a report on the first wave of CHNAs (22), but the outcome was a description of types of needs identified, not action plans. Select research studies have examined how dollars have been spent. No thorough analysis of the impact of the regulatory policy itself has been conducted by government or private researchers.

A recent special issue of *Frontiers in Public Health Education and Promotion* on Implementation Science pertaining to public health includes several articles that expand upon the need for precision in articulating relationships of both actors to action and actions to outcomes for community-oriented activities that include multiple organizations. The likelihood of successfully attaining the desired outcomes and of the collaborative partnership arrangements being deemed a success, and therefore sustained, warrant clarity at the outset. In “From Classification to Causality” Lewis and colleagues (23) capture the fundamental challenge with the IRS approach to Schedule H, arguing that successful interventions should be built upon causal pathways, which themselves should be based in theory as well as observational outcomes. Huynh et al. emphasize the need for complex analyses that dissect the multiple factors that contribute to the outcomes of complex problems, such as those comprising the health status of a given community (24). Huang et al. (25) discuss the impact of partnerships on interventions. At the same time the field is pushing collaborations because of the increased recognition of SDOH, how can a single hospital take full credit for the results of an intervention? All of these studies suggest that the relationships between a hospital's activities and the health of its community are multi-faceted and complex. A simple reporting form that shows dollars allocated according to categories determined by tax forms is inadequate to indicate a valid measure of an organization's impact on a diverse and arbitrary or amorphous target.

In the decade since the passage and roll-out of the ACA, no causal pathway or theoretical model guiding the evolution of the CB requirement or measuring its impact as a policy has emerged. The presumed goal of improving the health of a community has not included objectives or measures reflecting the consensus of a given community. Compiling existing research allows us to synthesize the current state of the art and outline what should be done for the future to evaluate the impact of this policy on the nation's health. For the contribution of hospitals of all types to their communities to be evaluated and measured, precise organizational models or causal paths, supported by theory, must be established, adequate time allowed for impact to occur, expectations must be set out in advance, and precision must be used in measuring results based on metrics that are feasible to gather and for which the professional and lay communities agree that the driving activities have produced the changes.

Until community benefit evolves to the point where the definition of the community is not the purview of the individual hospital, the indicators of the community's health status are determined by national consensus and set as goals to be achieved through measurable objectives, and hospitals face penalties for failure to comply with the process and achievement of set target outcomes, the effectiveness of the IRS reporting requirement remains questionable policy. Moreover, as long as the vast majority of community benefit funds are devoted to charity care and uncompensated care, and the remainder spread across a variety of programs attempting to meet multiple community needs, the likelihood of any given activity changing the health status of the community in a measurable way is slim.

## Way Forward

**Figure 3:**
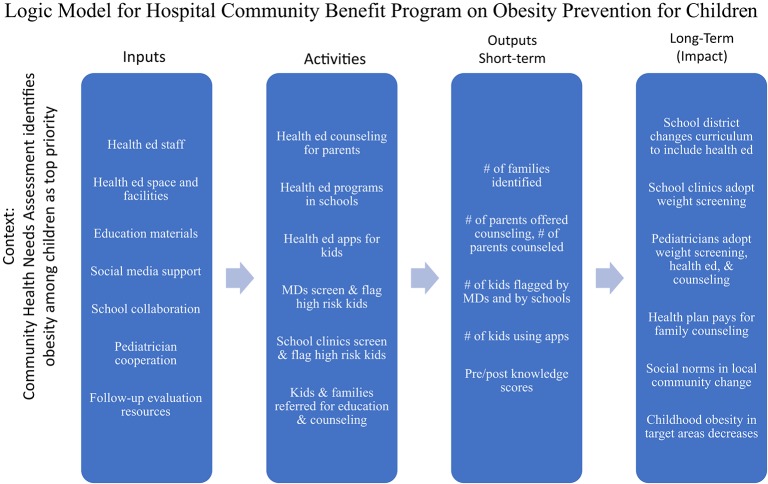
Logic model for community benefit program orchestrated by a single hospital.

In the absence of guidance from the federal regulatory agencies, non-profit hospitals must continue to submit Form 990 Schedule H and act with sufficient commitment to avoid any penalties that might be forthcoming in the future. Meanwhile, millions of dollars have been and are being spent in hopes of improving the health status of communities. Each hospital can take upon itself the obligation and opportunity to channel its activities in ways that are consistent with its missions and that make a documentable difference. Activities conducted under the auspice of CB, or with funds allocated to CB, should be selected from evidence-based programs and evaluated with specific measures. An example of a logic model for a program initiated by a single hospital to decrease obesity among its community members is included in the Figure 3. This type of discipline should be used in structuring a CB program. This does not necessarily imply additional resources or increased burden of reporting, but rather, careful selection of what actions are taken and how.

Programs done in collaboration with other community organizations should also use an evidence-based approach to planning and implementation, and evaluation should be sufficiently rigorous and detailed to consider the environment and the contribution of individual organizations as well as the collective. Changes in the health status of a community take time and require evaluations appropriate for measuring long-term outcomes and impact. Institutional commitment must blend long-range perspective and resource commitment with short-term demands for regulatory reporting.

Going forward, we suggest the following approach:

For healthcare executives and institutions:

- Take the CHNA seriously, as evidenced by the consistency with mission, engagement of governance, management, and operation in response to the identified needs of the community.
- Prioritize areas where evidence-based programs offer confidence that interventions selected to be used have been proven to be effective in a similar context.
- Select measures of health status that are realistic, useful, available, and that can be tracked over time. Be realistic about the potential of measurable outcomes for programs with small Ns.
- Perform the required evaluations rigorously, with fewer done better rather than many done superficially.
- Engage the appropriate expertise at all phases; build awareness and capacity internally.
- Be cognizant of all the other factors and other organizations that might affect an intervention, positively and negatively.
- Don't over-promise to the community, board and other stakeholders.
- Report change frequently and accurately, to both internal and external stakeholders.

For policy-makers and researchers:

- Policy analysts should advocate for an evaluation of the CB reporting requirements to determine the costs and benefit of this regulation.
- The US Department of Health and Human Services should negotiate with the IRS to take responsibility for advancing and monitoring the implementation of the community benefit requirement.
- Schedule H should be revised to relate activities directly to measures of benefit to the community, including measures of health status, and to recognize the respective allocation of funds to allowable categories other than charity care and uncompensated care.
- The methodology for evaluating projects done using the Collective Impact approach should be refined to allow the results of the operations and contributions of individual institutions to be distinguished from the results of the collaborative effort.
- For-profit hospitals and government hospitals should be asked to contribute to the health of the communities they serve, independent from the IRS regulatory requirement for non-profit hospitals.
- The metrics and methods for measuring the health status of a community should be refined to enable consensus on accurate, efficient and time-sensitive measurements that can be used by all organizations in the community.

Non-profit hospitals are currently spending millions of dollars on activities counted by the IRS as “community benefit.” A clear relationship between the activities undertaken by non-profits and measurable improvement in the health status of a given community is a worthy goal. Funding of community activities by hospitals of all types is to be encouraged, and removing the constraints forced by the regulation might improve rather than discourage hospital-community collaboration.

## Conclusions

The purpose of this article is to put community benefit in perspective as a national policy warranting evaluation and to encourage actions by individual non-profit hospitals and health systems to implement required regulations within a framework that provides evidence of impact at the local level. At present, spending on community benefit might not represent the best use of scarce healthcare resources because no one can measure the outcome of the activities being funded as a result of the IRS specifications.

Hospitals ask the IRS, “Does this count?” “Are we doing enough?” We cannot answer these questions until the assumptions are examined and the expectations expressed as goals with measurable objectives. Only by taking the next step of rigorous evaluation mapped to specific objectives and long-term goals can we have hope that the myriad activities being implemented across the nation under the guise of community benefit will actually benefit the community.

## Author Contributions

The two authors wrote this jointly. CE and PJ contributed to the conceptualization, and reviewed and edited the final version of the manuscript. CE wrote the first draft. PJ revised and refined.

### Conflict of Interest

The authors declare that the research was conducted in the absence of any commercial or financial relationships that could be construed as a potential conflict of interest.
